# Long-term impact of a school-based nutrition intervention on home nutrition environment and family fruit and vegetable intake: A two-year follow-up study

**DOI:** 10.1016/j.pmedr.2020.101247

**Published:** 2020-11-18

**Authors:** Allison N. Marshall, Christine Markham, Nalini Ranjit, Gregory Bounds, Joanne Chow, Shreela V. Sharma

**Affiliations:** a Michael & Susan Dell Center for Healthy Living, The University of Texas Health Science Center School of Public Health, 1616 Guadalupe, Austin, TX 78701, United States; b Center for Health Promotion and Prevention Research, The University of Texas Health Science Center-School of Public Health, Houston, 7000 Fannin St, Houston, TX 77030, United States; c Michael & Susan Dell Center for Healthy Living, The University of Texas Health Science Center-School of Public Health, 1200 Pressler, Houston, TX 77030, United States

**Keywords:** School-based intervention, Fruit and vegetable intake, Low-income populations, Dietary behavior, Sustained impact

## Abstract

Long-term data on maintenance of intervention effects of health promotion programs targeting fruit and vegetable (F&V) intake in children is lacking. We conducted a two-year follow-up of Brighter Bites, a school-based nutrition education and food co-op intervention found to be effective in increasing child intake of F&V. A one-group, pre-post evaluation design was used to assess the two-year post intervention impact of the program on child and parent dietary intake and home nutrition environment. In 2016–2017 school year, we conducted a follow up of 262 parent-child dyads who had previously participated in Brighter Bites in a 2013–2015 evaluation study in six low-income Texas elementary schools. Child dietary intake was measured using a parent-reported food frequency questionnaire, and surveys measured parent F&V intake, and home nutrition environment. Results of a multi-level regression analysis showed that, two years post-intervention, as compared to baseline, there was a significant increase in child intake of fruit, vegetable, and fiber, and significant decreases in total fat intake and percent daily calories from sugary beverages (p < 0.05). Parent dietary data showed significant increases in fruit intake, and intake of F&V combined (p < 0.05). Changes in home nutrition environment included: increased frequency of cooking behaviors, increased usage of nutrition facts labels in making grocery purchasing decisions, and increased food availability of F&V (p < 0.05). This study demonstrates potential long-term sustained impact of a comprehensive school-based intervention among low-income children and their families.

## 1 Introduction

### 1.1 Background

Adequate fruit and vegetable (F&V) intake is critical for proper child growth and development (US Department of Health and Human Services and U.S. Department of Agriculture., 2015, Ogden et al., 2010, World Health Organization. Global strategy on diet, physical activity, and health: promoting fruit and vegetable consumption around the world. http://www.who.int/dietphysicalactivity/fruit/en/. Updated, 2013). Even with health promotion efforts, F&V intake remains below recommendations across age groups in nationwide surveillance conducted as part of the National Health and Nutrition Examination Survey (NHANES) (US Department of Health and Human Services and U.S. Department of Agriculture, 2015). Moreover, children from lower socioeconomic households are likely to consume fewer F&V than higher socioeconomic households (Lee-Kwan et al., 2017).

While health promotion programs have successfully demonstrated short-term impact on F&V intake among children, few studies report on long-term maintenance of effects on behaviors (Zarnowiecki et al., 2014, Jones et al., 2011). There is a need for long-term follow-up of dietary interventions among low-income populations to determine sustainability of intervention effects, and to further refine intervention strategies (Zarnowiecki et al., 2014, Jones et al., 2011, Appleton et al., 2016); supported by multiple systematic reviews (Jones et al., 2011, Appleton et al., 2016).

Brighter Bites is a 16-week school-based nutrition intervention targeting increased F&V intake among low-income children and families. A two-year quasi-experimental non-randomized controlled study conducted in 2013–2015 to assess the impact of Brighter Bites among 1st grade children across one school year demonstrated significant improvements in child dietary intake and home nutrition environment among participating families compared to those in wait-list comparison schools (Wang and Stewart, 2013). Subsequently, we present results of a two-year follow-up study in 2016–2017 to determine program maintenance effects.

## 2 Methods

### 2.1 Design

**Fig. 1:**
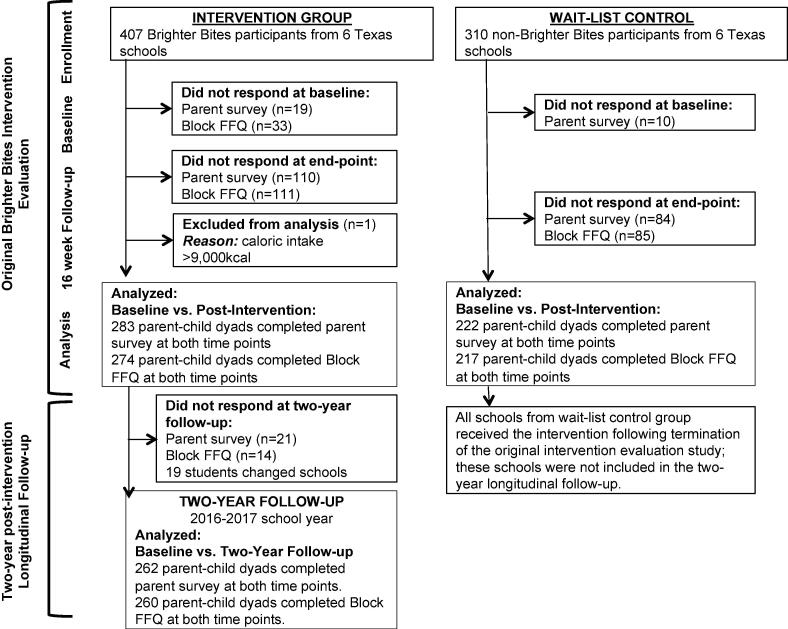
Study Flow of a Two-Year Longitudinal Follow-up Evaluation of Brighter Bites.

We conducted a two-year follow-up using a one-group pre-post evaluation design across the six intervention schools that participated in Brighter Bites in the 2013–2015 evaluation study. Control schools from the parent study could not be included in the two-year follow-up; waitlist controls received the intervention at the end of 2015 (see Fig. 1).

#### 2.1.1 Description of brighter bites

Brighter Bites is a theory-grounded school-based health promotion program. Detailed description of Brighter Bites is provided elsewhere (Wang and Stewart, 2013). Briefly, Brighter Bites intends to increase F&V demand and consumption by children and parents through a 16-week school-based food co-op during one school year offering weekly fresh produce distribution (~50 servings/family); nutrition education in schools and for parents; and weekly recipe tastings during produce pick up time. Brighter Bites has a 3-on 3-off formula whereby they are in a school three years in a row (assuming the school chooses to continue). This allows families sustained access to the program for up to three years if they choose to continue. Brighter Bites is implemented in early childhood centers and elementary schools, typically serving children ages 3–12 years old and their families.

### 2.2 Participants

In the parent study in 2013–2015 (Wang and Stewart, 2013); six intervention schools received Brighter Bites for one school year (n = 407 parent-child dyads); six comparison schools implemented an evidence-based coordinated school health program (n = 310 parent-child dyads) in Houston, Texas (see Fig. 1). A convenience sample of public and charter schools that enrolled 1st grade children with >75% of children enrolled in the free/reduced lunch program were eligible to participate in the parent study. Within each school, two to three 1st grade classrooms were targeted. Consent packets were sent home to all parents in selected classrooms; all students were eligible to participate in Brighter Bites, but only those who consented were measured. Pre-/post evaluation was conducted across intervention and comparison schools at baseline and end of one school year (Wang and Stewart, 2013). In the 2016–2017 school year, we conducted a two-year follow-up of families in intervention schools only. At this time, all schools in the parent study were receiving the Brighter Bites program. All participants completing baseline and post-intervention measurements in the parent study were eligible for inclusion in this follow-up study (completers). Once school consent was obtained for the follow-up study, child participant school and grade information from the parent study was used to send home consent packets and study surveys with children to parents. Of 407 families participating in Brighter Bites who completed baseline data in the parent study, there were 283 completers (69.5% of families completing baseline) at the end of 2013–2015 parent study, of which 262 parent-dyads (92.6% of completers) agreed to participate in this two-year follow-up study. Respondents to the follow-up study were more likely to be older (*p =* 0.03), mother in relationship to child (*p* = 0.001), Hispanic (*p* = 0.001), born in a country other than the U.S. (*p* < 0.001), and bilingual (*p* = 0.001) (data not presented in tables). Study flow is presented in Fig. 1. Written informed consent was obtained from all parents. The study was approved by the University of Texas Health Science Center, Committee for Protection of Human Subjects.

### 2.3 Measures

Follow-up measurements were obtained in Spring 2017 using the same measures as the parent study (Wang and Stewart, 2013).

#### 2.3.1 Child dietary intake

Child dietary intake was measured using the parent-reported, previously validated Block Kids Food Screener (BKFS) which includes 41 items to assess both frequency and quantity of foods consumed (Sharma et al., 2016). The BKFS was sent home with children, self-completed by parents, and returned to project staff through children. Completed measures were sent to Nutrition Quest for analysis.

Responses were used to assess number of servings of various foods consumed, including fruit and fruit juices, vegetables, potatoes (including French fries), whole grains, meat/poultry/fish, dairy, legumes, saturated fat (calculated from intake of each FFQ line item aside from sodas), and “added sugars” (calculated from intake of sweetened cereals, soft drinks, and sweets) (Sharma et al., 2016). All portion sizes are age and sex-specific. Dietary intake data were calculated in nutrient densities and divided by total caloric intake to standardize all dietary intake data to 1000 kcal to adjust for increase in caloric intake over time, which is expected as children grow, and to allow for comparability within and between subjects. Intake of sugary beverages was determined (both kcal and frequency).

#### 2.3.2 Parental F&V intake

Parental F&V intake was measured using a previously validated 10-item self-report Fruits and Vegetable Screener National Institutes of Health (NIH) block screener (Hunsberger et al., 2015). Frequency of F&V consumption was assessed over the past month with 9 response options (never to 5 or more times per day). This was scored using National Cancer Institute calculation protocols (linked to MyPyramid guidelines); intake is presented in cups (Thompson et al., 2002).

#### 2.3.3 Home nutrition environment

Parental rules for limiting portion sizes, screen time, fried foods, fast food, and sugary beverages, and eating family dinners and rules to finish all foods on plates were measured using a self-report questionnaire of items previously validated with similar populations (National Institutes of Health National Cancer Institute Division of Cancer Contol Population Sciences. Fruit vegetable intake screeners in the eating at America's table study (EATS): Scoring the all-day screener. Epidemiology and Genomics Research Program: Research Resources Web site. https://epi.grants.cancer.gov/diet/screeners/fruitveg/scoring/allday.html. Updated Feb 11, 2020, Ding et al., 2012, Edmundson et al., 1996, Baranowski et al., 2000). Home mealtime environment was assessed using previously validated items for frequency of cooking from scratch at home, eating out, using nutrition facts labels, F&V served at mealtimes and snacks, sugary cereals, and sugar-sweetened drinks at meals (Ding et al., 2012, Edmundson et al., 1996, Baranowski et al., 2000). These items used a Likert-type scale.

### 2.4 Statistical analyses

All analyses were performed using STATA software, version 14.2. Means, standard deviations (SD) and frequencies were computed for all demographic data and other variables of interest. Repeated measures mixed effects linear regression models were applied to account for clustered data with time (level 1) nested in subjects (level 2), and school-level clustering. All macro and micronutrients were standardized to 1000 kcal/day. Standardizing intake to 1000 kcal/day adjusts for the increase in intake over time and allows for comparability of nutrients across time periods. Changes in child F&V intake, parent F&V intake, and home nutrition environment from baseline (2013–2014) to two-year post-intervention follow-up (2016–2017) were estimated. Socio-demographic variables were included in models only if coefficients were changed by >10%. Significance was at p < 0.05. Additionally, we adjusted for Brighter Bites attendance among families in all models. Missing data are likely not random, Maximum Likelihood Estimate (MLE) was used in analyses but we did not use any imputations because <10% of data was missing (Penkilo et al., 2008).

## 3 Results

### 3.1 Participants

At baseline in 2013–2014 (n = 407), 55.9% of households were bilingual, 53.8% of child participants were girls and 44.0% of children were overweight or obese. Child participants were 5–7 years old (mean = 6.12, SD = 0.34), 75.5% of parents were Hispanic, and 21.3% were African American. Most parents were mothers of participating children (92%), with 19 fathers (7.6%) and 1 grandmother (0.4%). The average household size was 5.28 (SD = 5.73).

### 3.2 Changes in child dietary intake

At two-year post-intervention follow-up, as compared to baseline, there was a significant increase in child intake of fruits (+0.18 cups/1000 kcal; β = 0.16, 95% CI: 0.05, 0.27, p ≤ 0.01) and vegetables (+0.14 cups/1000 kcal; β = 0.14, 95% CI: 0.09, 0.19, p ≤ 0.001) (Table 1). As compared to baseline, child consumption of fiber at two year post-intervention follow-up also increased significantly (+1.06 g/1000 kcal/day; β = 0.97, 95% CI: 0.56, 1.38, p < 0.001) and consumption of total fat in grams per 1000 kcal decreased significantly (−1.55 g/1000 kcal; β = −1.30, 95% CI: −2.20, −0.41, p < 0.01). Child consumption of added sugar decreased significantly from baseline to follow-up (β = −0.63, 95% CI: −1.00, −0.27, p = 0.001); additionally, there was a significant decrease in percent calories consumed from sugary beverages (−0.52%; β = −0.61, 95% CI: −2.24, −0.09, p = 0.022) from baseline to follow-up. The average number of calories consumed increased significantly from baseline to follow-up (+110.72 kcal; β = 115.8, 95% CI: 8.68, 222.91, p = 0.03), which is expected as children grow. Interestingly, child consumption of potatoes and French fries increased significantly from baseline to follow-up (+0.03 cups/1000 kcal; β = 0.03, 95% CI: 0.008, 0.05, p < 0.01).

**Table 1:** Changes in variables targeted in Brighter Bites: child and parent dietary intake, parental food practices, rules and home nutrition environment from baseline to two-year follow-up, Brighter Bites 2016–2017, central Texas n = 260 parent child dyads for child block FFQ, n = 262 parent child dyads for parent survey).

	Baseline	Final	Mixed Effects Models	
	mean(SD)	mean(SD)	β^α^(95% CI ^λ^)	P-value
*Child Block Dietary Data (N = 260)*				
**Fruits** (cup/1000 kcal/day)	1.22(0.81)	1.40(0.74)	0.16 (0.05, 0.27)	<0.01*
**Vegetables** (cup/1000 kcal/day)	0.58(0.35)	0.72(0.38)	0.14 (0.09, 0.19)	<0.0001*
**Added Sugar** (tsp/1000 kcal/day)	5.36(2.84)	4.77(2.50)	−0.63 (−1.00, −0.27)	0.001*
**Estimated percent of daily kcal from sugar beverages^a^** (%)	2.90(4.31)	2.38(3.24)	−0.61 (−1.13, −0.09)	0.02*
**Total Fiber** (grams per 1000 kcal/day)	9.94(3.24)	11.00(3.31)	0.98 (0.57, 1.39)	<0.001*
**Total Fat** (grams per 1000 kcal/day)	39.28(6.84)	37.73(5.18)	−1.31 (−2.20, −0.41)	<0.01*
**Average Daily Kilocalories** (kcal per day)	1089.93(610.47)	1200.65(772.15)	115.11 (7.88, 222.34)	0.04*
**Potatoes, including French Fries** (cup/1000 kcal/day)	0.20(0.16)	0.23(0.14)	0.03 (0.01, 0.05)	<0.01*
**Whole grains** (ounce/1000 kcal/day)	0.48(0.33)	0.49(0.32)	0.02 (−0.03, 0.07)	0.52

*Parent Survey Fruits and Vegetables Screener (N = 262)*				
**Fruit Group^γ^**	1.79(2.17)	1.93(2.25)	0.04 (−0.08, 0.16)	0.52
**Vegetable Group^δ^**	1.37(1.96)	1.97(2.87)	0.20 (0.07, 0.33)	<0.01*

**Fruits and Vegetables combined^ε^**	3.16(3.7)	3.92(4.4)	0.24 (0.03, 0.46)	0.03*
	**Baseline**	**Final**	**Mixed Effects Models**	
	n(%)	n(%)	β^α^ (95% CI ^λ^)	P-value

*Parental food practices*				
**How often do you understand the Nutrition Facts Table on fruit and drink packages?**				
Always/ often	93(37.7)	117(49.8)	0.27 (0.15, 0.38)	<0.001*
Sometimes	64(25.9)	67(28.5)		
Never/ Rarely	90(36.4)	51(21.7)		
**Use the Nutrition Facts Table on food and drink help you with your purchase decision?**				
Always	30(12.3)	37(15.8)	0.46 (0.29, 0.64)	<0.001*
Often	46(18.8)	75(31.8)		
Sometimes	53(21.7)	58(24.8)		
Rarely	78(32.0)	44(18.7)		
Never	37(15.2)	21(8.9)		
**Cook from scratch at home, using fresh/frozen ingredients food?**				
Once per day or more often	119(48.6))	115(48.9)	1.05^ϕ^ (0.67, 1.63)	0.83
Less than once per day	126(51.4)	120(51.1)		
**In the past week, how many times did you eat food from any type of restaurant?**				
Everyday	5(2.0)	2(0.9)	−0.27 (−0.38, −0.16)	<0.001*
5–6 times	6(2.4)	0(0.0)		
3–4 times	30(12.1)	16(6.8)		
1–2 times	160(64.5)	141(59.7)		
Never	47(19.0)	77(32.6)		

*Home mealtime environment (N = 262)*				
**During the past 7 days, how many times:** **Were fresh/frozen fruits served as snacks to your child in your home?**				
Everyday	45(18.3)	45(19.2)	0.11 (−0.06, 0.29)	0.21
5–6 times	24(9.7)	31(13.2)		
3–4 times	72(29.3)	63(26.7)		
1–2 times	64(26.0)	66(28.1)		
Never	41(16.7)	30(12.8)		
**Were fresh/frozen vegetables served to your child at evening meal in your home?**				
Everyday	31(12.6)	51(22.0)	0.43 (0.24, 0.62)	<0.001*
5–6 times	36(14.6)	39(16.8)		
3–4 times	61(24.7)	64(27.6)		
1–2 times	84(34.0)	59(25.4)		
Never	35(14.2)	19(8.2)		
**Were 100% whole-wheat or whole-grain bread or tortillas served to your child at meals in your home?**				
5 times or more	73(29.5)	74(31.7)	0.04 (−0.05, 0.14)	0.38
1–4 times	137(55.5)	130(55.8)		
Never	37(15.0)	29(12.5)		
**Were sugar sweetened cereal served to your child at breakfast in your home?**				
5 times or more	52(21.2)	28(12.0)	−0.15 (−0.25, −0.06)	0.002*
1–4 times	158(64.2)	157(67.1)		
Never	36(14.6)	49(20.9)		
**Were sugar sweetened drinks served at the evening meal in your home?**				
5 times or more	40(16.3)	22(9.4)	−0.16 (−0.24, −0.07)	<0.001*
1–4 times	152(61.7)	139(59.2)		
Never	54(22.0)	74(31.4)		
**Did your child help you prepare your evening meal?**				
Everyday	15(6.2)	12(5.2)	0.18 (0.04, 0.33)	0.02*
5–6 times	6(2.5)	7(3.0)		
3–4 times	18(7.5)	35(15.0)		
1–2 times	94(39.0)	102(43.7)		
Never	108(44.8)	77(33.1)		

*Parental rules (N = 262)*				
**Do you have the following rules about your child’s eating?** **Limit portion sizes?**				
Yes	118(48.7)	105(44.7)	1.43^ω^ (0.99, 2.06)	0.05
Sometimes	58(24.0)	81(34.4)		
No	66(27.3)	49(20.9)		
**No meals while watching TV/DVDs?**				
Yes	81(33.5)	75(31.9)	1.19^ω^ (0.81, 1.74)	0.37
Sometimes	83(34.3)	88(37.5)		
No	78(32.2)	72(30.6)		
**No fried snacks (such as potato chips) at home?**				
Yes	38(15.7)	43(18.4)	0.83^ω^ (0.58, 1.18)	0.30
Sometimes	123(50.6)	109(46.6)		
No	82(33.7)	82(35.0)		
**Must eat dinner with the family?**				
Yes	170(69.6)	149(63.4)	1.48^ω^ (0.95, 2.29)	0.08
Sometimes	47(19.3)	58(24.7)		
No	27(11.1)	28(11.9)		
**Limit fast food?**				
Yes	174(71.0)	165(69.9)	1.16^ω^ (0.74, 1.82)	0.52
Sometimes	48(19.6)	50(21.2)		
No	23(9.4)	21(8.9)		
**No sugary beverages?**				
Yes	95(38.8)	96(40.8)	0.91^ω^ (0.62, 1.34)	0.63
Sometimes	95(38.8)	86(36.6)		
No	55(22.4)	53(22.6)		
**Must finish all food on plate?**				
No	49(19.8)	54(23.1)	1.05^ω^ (0.72, 1.53)	0.81
Sometimes	74(30.0)	69(29.5)		
Yes	124(50.2)	111(47.4)		

α Coefficients were calculated using Multilevel Mixed Effects Models.

λ CI stands for confidence interval.

γ Total daily number of MyPyramid servings for fruits which includes consumption of 100% juice, fresh, canned, and frozen fruits and excludes fruit drinks like Kool-Aid, lemonade, Hi-C, Tang, and Twister. My Pyramid defines servings in cup equivalents with 1 cup of fruit, 100% fruit juice, or ½ cup of dried fruit as 1 cup equivalent. (Graham, 2009).

δ Total daily number of MyPyramid servings for vegetables which includes consumption of lettuce salad, tomato sauce, vegetable soups and other vegetables which excludes white potatoes, cooked dried beans, and vegetables in mixtures. MyPyramid defines servings in cup equivalents with 1 cup of raw, cooked, or canned vegetables; 2 cups of raw leafy green vegetables; and ½ cup dried vegetables as 1 cup equivalent (Graham, 2009).

ε Sum of total daily number of MyPyramid servings for fruits and vegetables.

ϕ Odds ratios were calculated using Mixed Effects Logistic Regression.

ω Odds ratios were calculated using Multilevel Mixed Effects Ordered Logistic Regression.

* Findings statistically significant at P ≤ 0.05.

^a^ Added sugar is based on consumption of sweetened cereals, soft drinks, and sweets based on the Block Kids Food Screener.

### 3.3 Changes in parental dietary intake

As compared to baseline, at two years follow-up, parents reported significant increases in daily intake of vegetables (+0.6 cups; β = −0.20, 95% CI: 0.07, 0.33, p ≤ 0.01), and combined F&V (β = 0.24, 95% CI: 0.03, 0.46, p = 0.03). Upon further exploration, these increases in vegetable intake were primarily from increased intake of lettuce salads (+0.17 cups; β: 0.06, 95% CI: 0.02, 0.09, p < 0.01) and ‘other vegetables’ (+0.30 cups, β: 0.10, 95% CI: 0.03, 0.18, p < 0.01) which includes all raw, cooked, canned, and frozen vegetables aside from lettuce salads, white potatoes, cooked dried beans, rice, vegetables in mixtures, such as in sandwiches, omelets, casseroles, Mexican dishes, stews, stir-fry, soups, etc. (Hunsberger et al., 2015).

### 3.4 Home nutrition environment

Changes in home nutrition environment also persisted in a two-year follow-up after post intervention measurement in Spring 2015 (Wang and Stewart, 2013). From baseline to two-year follow up, parents reported significant decreases in frequency of eating out (β = −0.27, 95% CI: −0.38, −0.16, p ≤ 0.001), increased understanding of nutrition facts labels (β = 0.27, 95% CI: 0.15, 0.38, p ≤ 0.001), and increased frequency of using nutrition facts labels to make food-purchasing decisions (β = 0.46, 95% CI: 0.29, 0.64, p ≤ 0.001). There were also significant increases in children eating breakfast daily (OR: 0.55, 95% CI: 0.33, 0.92, p = 0.02), child participation in evening meal preparation (β = 0.18, 95% CI: 0.04, 0.33, p = 0.01), and serving of fresh or frozen vegetables to children at evening meals (β = 0.43, 95% CI: 0.24, 06.2, p < 0.001). Significant decreases were found in frequency of serving of sugar-sweetened cereal to children at breakfast (β = −0.15, 95%CI: −0.25, −0.06, p = 0.002), and serving of sugar-sweetened drinks at evening meals (β = −0.15, 95%CI: −0.24, −0.07, p < 0.001).

## 4 Discussion

Our study adds to current literature assessing maintenance of intervention effects among low-income children and families. We saw maintenance of many post-intervention behavior and home environmental changes at two-year follow up, including child F&V intake, decreased child consumption of added sugars, and parental understanding and usage of nutrition labels to make food purchasing decisions (Wang and Stewart, 2013). Moreover, we saw additional positive behavior changes in parent intake not evident immediately post-intervention, including increased intake of vegetables, and decreased frequency of eating out (Wang and Stewart, 2013). Prior studies and theoretical models have demonstrated that parent behavior is an important mediator for change in child F&V intake (Rasmussen et al., 2006). Those that incorporate strategies of F&V provision coupled with education could have long-term impacts on both parent and child diet.

Our study adds to current literature on longer-term intervention effects for children and parents. In a 2016 systematic review, Appleton et al. found that long-term effects are often not assessed, making it challenging to determine maintenance of intervention effects (Jones et al., 2011). Prior studies have identified barriers to purchasing produce among low-income families including cost, transportation, and lack of quality and variety available (Haynes-Maslow et al., 2013). One likely reason for maintenance effects seen in our study is because programs such as Brighter Bites use access plus education strategies whereby low-income families get a substantial amount of produce to take home regularly while learning how to use it, thus creating health habits. Studies show both access and education are important to achieve behavior change (Verghese et al., 2019). Families on a limited budget may not purchase or consume F&V due to lack of access, affordability, or knowledge on how to use it, or fear that their family may not consume it (Drewnowski and Darmon, 2005, Hilmers et al., 2012), thus effectively reducing demand for F&V over time in these families. With Brighter Bites, families get a free trial of 20–25 lbs. of 8–12 different kinds of produce weekly for 16 weeks in a school year while learning how to use it, allowing low-income families with children to consume F&V without financial risk while they are in the program. Brighter Bites has a three on three off formula whereby they are in a school three years in a row (assuming the school chooses to continue). This allows families sustained access to the program for up to three years if they choose to stay in the program*.* Furthermore, evidence suggests that children need to try new foods 12–14 times before liking it (Birch and Fisher, 1998), which is reinforced in Brighter Bites. Our study demonstrates that these strategies are potentially creating a longer-term demand for F&V such that families may continue these habits by obtaining F&V even after the program ends.

### 4.1 Strengths

Strengths of the study include follow-up time to evaluate sustained intervention effects. Additionally, this study measures both child and parent diet; includes measures of home nutrition environment; and is focused on a low-income sample. Finally, the response rate of our sample was relatively high (92.5%).

### 4.2 Limitations

This study relies on self-report of dietary intake and home nutrition environment. Without a comparison group, it is not possible to determine if observed changes are solely attributable to the Brighter Bites intervention; estimates may be inflated due to selection bias due to attrition. Missing data are likely not random, MLE was used in analyses; we did not use any imputations because <10% of data was missing. We did not have a comparison group for follow-up; it received Brighter Bites after the parent study period. However, changes seen in the current follow-up study suggest maintenance of intervention effects among those who participated in the long-term follow-up. Given self-report data, there is social desirability bias; we expect this would be consistent across time-points and should not affect the point estimate. These limitations notwithstanding, our study demonstrates potential sustained positive longer-term impacts of a comprehensive school-based intervention among low-income children and families.

## CRediT authorship contribution statement

**Allison N. Marshall:** Conceptualization, Data curation, Visualization, Writing - review & editing. **Christine Markham:** Conceptualization, Methodology. **Nalini Ranjit:** Methodology, Writing - review & editing. **Gregory Bounds:** . **Joanne Chow:** Methodology. **Shreela V. Sharma:** Conceptualization, Methodology, Writing - review & editing, Supervision.

## Declaration of Competing Interest

Dr. Sharma is on the Board of Directors of Brighter Bites nonprofit organization, the goal of which is to improve access to fresh fruits and vegetables and nutrition education among underserved communities. This is an unpaid, advisory board position. The other authors have no conflicts of interest relevant to this article to disclose.
